# Prefrontal cortical modulation of information flow in a large-scale model of the cortico-thalamic circuit

**DOI:** 10.1186/1471-2202-14-S1-P410

**Published:** 2013-07-08

**Authors:** Riccardo Zucca, Paul FMJ Verschure

**Affiliations:** 1 Laboratory of Synthetic, perceptive, emotive and cognitive systems (SPECS), DTIC, Universitat Pompeu Fabra, Barcelona, Spain; 2 Institució Catalana de Recerca I Estudis Avançats (ICREA), Barcelona, Spain

## 

While generating the stream of consciousness and driving our actions in the world the brain largely relies on implicit forms of information processing. Conscious and unconscious factors are closely coupled and can be seen as complementary since unconscious processing can be sensitive to regularities within signals prior to conscious awareness, suggesting that the content of consciousness can be biased by unconscious factors.

In the context of the Distributed Adaptive Control (DAC) theory of the mind, brain and body nexus [1], we previously developed different probabilistic models of perception and attention that include bottom-up and top-down processes in defining an anticipatory gate to restrict the prediction of future incoming stimuli [2]. These results suggest that unconscious information presented at the border of this anticipatory gate could lead to a re-organization of perception and cognition by modifying sensory expectations. A plausible neuronal substrate for this top-down shaping mechanism has been identified in the circuitry including prefrontal cortical areas and the thalamus by mediation of the inhibitory thalamic reticular nucleus (TRN) interposed between the thalamus and the cortex. TRN is uniquely innervated by prefrontal projections, both by large ('driving') and small ('modulatory') synaptic boutons, that extends into the sensory sectors of the nuclei [3] indicating a possible role in the regulation of the sensory information flow from the thalamus to the cortex.

Building on an initial large scale model of thalamo-cortical dysrhythmia [4], the present study describes a spiking model of the cortico thalamic loops that aims at explaining how concurrent sensory inputs can be effectively gated by the activation of prefrontal projections to the TRN. The model consists of a network of pyramidal cells, interneurons, thalamic reticular and thalamocortical relay cells and includes driving and modulatory connections from the prefrontal areas to the TR nuclei. We show that inputs from the prefrontal cortex (and its homologous thalamic area) can selectively favor one of two incoming thalamic stimuli by activating neighboring TRN neurons and thus inhibiting the thalamic relay cells activated by the concurrent stimulus. The stimulus is then not sufficiently strong to be transmitted to the cortical areas. PFC top-down expectations can thus act as a filter on the thalamus (by the mediation of the RTN) to suppress irrelevant signals and maintain a coherent internal state. As a follow up we want to test the prediction that the same mechanism can drive to conscious percepts following strong expectations even in the absence of external stimuli.
